# Physiologic upper limit of pore size in the blood-tumor barrier of malignant solid tumors

**DOI:** 10.1186/1479-5876-7-51

**Published:** 2009-06-23

**Authors:** Hemant Sarin, Ariel S Kanevsky, Haitao Wu, Alioscka A Sousa, Colin M Wilson, Maria A Aronova, Gary L Griffiths, Richard D Leapman, Howard Q Vo

**Affiliations:** 1 National Institute of Biomedical Imaging and Bioengineering, National Institutes of Health, Bethesda, Maryland 20892, USA; 2 Radiology and Imaging Sciences Program, Clinical Center, National Institutes of Health, Bethesda, Maryland 20892, USA; 3 Imaging Probe Development Center, National Heart, Lung, and Blood Institute, National Institutes of Health, Bethesda, Maryland 20892, USA

## Abstract

**Background:**

The existence of large pores in the blood-tumor barrier (BTB) of malignant solid tumor microvasculature makes the blood-tumor barrier more permeable to macromolecules than the endothelial barrier of most normal tissue microvasculature. The BTB of malignant solid tumors growing outside the brain, in peripheral tissues, is more permeable than that of similar tumors growing inside the brain. This has been previously attributed to the larger anatomic sizes of the pores within the BTB of peripheral tumors. Since in the physiological state *in vivo *a fibrous glycocalyx layer coats the pores of the BTB, it is possible that the effective physiologic pore size in the BTB of brain tumors and peripheral tumors is similar. If this were the case, then the higher permeability of the BTB of peripheral tumor would be attributable to the presence of a greater number of pores in the BTB of peripheral tumors. In this study, we probed *in vivo *the upper limit of pore size in the BTB of rodent malignant gliomas grown inside the brain, the orthotopic site, as well as outside the brain in temporalis skeletal muscle, the ectopic site.

**Methods:**

Generation 5 (G5) through generation 8 (G8) polyamidoamine dendrimers were labeled with gadolinium (Gd)-diethyltriaminepentaacetic acid, an anionic MRI contrast agent. The respective Gd-dendrimer generations were visualized *in vitro *by scanning transmission electron microscopy. Following intravenous infusion of the respective Gd-dendrimer generations (Gd-G5, N = 6; Gd-G6, N = 6; Gd-G7, N = 5; Gd-G8, N = 5) the blood and tumor tissue pharmacokinetics of the Gd-dendrimer generations were visualized *in vivo *over 600 to 700 minutes by dynamic contrast-enhanced MRI. One additional animal was imaged in each Gd-dendrimer generation group for 175 minutes under continuous anesthesia for the creation of voxel-by-voxel Gd concentration maps.

**Results:**

The estimated diameters of Gd-G7 dendrimers were 11 ± 1 nm and those of Gd-G8 dendrimers were 13 ± 1 nm. The BTB of ectopic RG-2 gliomas was more permeable than the BTB of orthotopic RG-2 gliomas to all Gd-dendrimer generations except for Gd-G8. The BTB of both ectopic RG-2 gliomas and orthotopic RG-2 gliomas was not permeable to Gd-G8 dendrimers.

**Conclusion:**

The physiologic upper limit of pore size in the BTB of malignant solid tumor microvasculature is approximately 12 nanometers. In the physiologic state *in vivo *the luminal fibrous glycocalyx of the BTB of malignant brain tumor and peripheral tumors is the primary impediment to the effective transvascular transport of particles across the BTB of malignant solid tumor microvasculature independent of tumor host site. The higher permeability of malignant peripheral tumor microvasculature to macromolecules smaller than approximately 12 nm in diameter is attributable to the presence of a greater number of pores underlying the glycocalyx of the BTB of malignant peripheral tumor microvasculature.

## Background

The blood-tumor barrier (BTB) of malignant solid tumor microvasculature is more permeable to macromolecules than the endothelial barrier of normal tissue microvasculature of the continuous type[1,2]. This hyper-permeability of malignant solid tumor microvasculature to macromolecules has been attributed to the local release of vascular permeability factor in tumor tissue[3,4]. The BTB of malignant solid tumors growing outside the brain in peripheral tissues and organs is typically more permeable than the BTB of similar malignant solid tumors growing in the brain[5,6]. Furthermore, when a malignant peripheral tumor, such as a breast cancer tumor, metastasizes to the brain, an ectopic site, the permeability of the BTB of the breast cancer tumor growing in the brain is lower than the BTB of the original tumor in breast tissue, the orthotopic site[5]. The brain tissue host site microenvironment lowers the permeability of the BTB of metastatic malignant peripheral tumors such that it approximates the permeability of the BTB of orthotopic brain tumors like malignant gliomas[7,8].

Various sizes of pores have been identified in the BTB of malignant solid tumor microvasculature, which is discontinuous[1]. These include trans-endothelial cell fenestrations, caveolae and vesiculo-vacuolar organelles (VVOs) within endothelial cells, and inter-endothelial cell gaps between endothelial cells[1,4,9-12]. Based on electron microscopy, the anatomic pore size of the fenestrations, caveolae, and VVOs of the BTB of both brain tumors and peripheral tumors have been reported to range between 40 nm and 200 nm in diameter[10,13,14]. In contrast, the pore size of inter-endothelial cell gaps within the BTB of both brain tumors and peripheral tumors is much larger. In the case of brain tumors, inter-endothelial cell gaps have been reported to range between 100 nm and 3000 nm in diameter[10,13] and in the case of peripheral tumors the gaps have been reported to range between 300 nm and 4700 nm[12]. Although the diameters of the trans-endothelial cell fenestrations, caveolae, and VVOs are smaller than those of the inter-endothelial cell gaps, these pores are more numerous than the inter-endothelial cell gaps in the BTB of brain tumors and peripheral tumors[4,9,10]. The higher permeability of the BTB of peripheral tumors compared to the BTB of brain tumors has been previously attributed to the presence of larger inter-endothelial gaps in the BTB of peripheral tumors[12,15].

The pore size within the BTB of malignant solid tumors has been previously probed *in vivo *with intra-vital microscopy after the intravenous infusion of particles in the nanometer size range labeled on the exterior with rhodamine, a cationic fluorescent dye[15,16]. Cationic particles are known to be toxic to the negatively charged glycocalyx[17,18], which is the fibrous carbohydrate layer that coats the luminal surface of endothelial cells[19]. As a result cationic particles have been shown to increase the permeability of the BTB by disrupting the glycocalyx of the BTB [20-22]. With intra-vital fluorescence microscopy the transvascular extravasation of cationic nanoparticles across the BTB of malignant tumor microvasculature has been visualized and it has been reported that the upper limit of pore size within the BTB of malignant brain tumors ranges between 7 nm and 100 nm, whereas that the upper limit of pore size within the BTB of peripheral tumors ranges between 200 nm and 1200 nm[15].

In the case of malignant brain tumors, we recently probed the upper limit of pore size within the BTB of orthotopic RG-2 rat gliomas with dynamic contrast-enhanced MRI using dendrimer nanoparticles labeled on the exterior with gadolinium (Gd)-diethyltriaminepentaacetic acid (DTPA), an anionic MRI contrast agent[22]. Based on this work, we reported that the upper limit of pore size within the BTB of orthotopic RG-2 rat gliomas *in vivo *was approximately 12 nm[22]. These previously reported findings suggest that the impediment to the transvascular extravasation of particles across the BTB of brain tumors is at the level of the glycocalyx that coats the surface of the pores in the BTB and is a "nanofilter" for the transvascular flow of particles across the BTB[23].

It is possible that the physiologic upper limit of pore size within the BTB of peripheral tumors previously reported as being between 200 nm and 1200 nm[15] may be a gross over-estimation of the actual physiologic upper limit of pore size within the BTB of peripheral solid tumors. Therefore, if the actual physiologic upper limit of pore size within the BTB of peripheral tumors is significantly lower than what has been previously reported, and approximates that of the BTB of brain tumors, then this finding would suggest that more pores in BTB of peripheral tumors are the primary reason for the higher permeability of the BTB of malignant peripheral tumors compared to that of malignant brain tumors. Furthermore, such findings would have important implications on the size range of therapeutics that could be effectively delivered across the BTB of malignant solid tumors independent of tumor host site.

In our previous dynamic contrast-enhanced MRI-based work[22], we had characterized the upper limit of pore size within the BTB of orthotopic RG-2 malignant gliomas using successively higher generation (G) polyamidoamine (PAMAM) dendrimers labeled with Gd-DTPA. With dynamic-contrast enhanced MRI, we found there to be significant positive contrast enhancement of brain tumor tissue following the intravenous infusion of Gd-G1 through Gd-G7 dendrimers, but not following the intravenous infusion of Gd-G8 dendrimers. Based on this observation, we established that Gd-G8 dendrimers were larger than the physiologic upper limit of pore size within the BTB of orthotopic RG-2 gliomas. With this dynamic contrast-enhanced MRI approach, in addition to being able to image the tumor tissue pharmacokinetics of Gd-G1 through Gd-G8 dendrimers, we were also able to image at the same time the blood pharmacokinetics of the respective Gd-dendrimer generations in the large vessels within the brain. We found that the higher generation Gd-G5 through Gd-G8 dendrimers maintained steady state blood concentrations over the 120 minute long imaging session. Since Gd-G5, Gd-G6, and Gd-G7 dendrimers maintained steady state blood concentrations over the 120 minute imaging session and were permeable to the BTB of orthotopic RG-2 brain tumors, these higher generation Gd-dendrimers continued to accumulate within the tumor tissue extravascular space over time, and remained there for sufficiently long to localize within individual glioma tumor cells. Although these imaging sessions were long enough to determine the physiologic upper limit of pore size in the BTB of orthotopic brain tumors as well as qualitatively assess the blood half-lives of lower generation Gd-dendrimers, we were unable to qualitatively assess the blood half-lives of the higher generation Gd-dendrimers, since the higher generation Gd-dendrimers maintained steady state blood concentrations over 120 minutes.

In present study, we imaged the blood and tumor tissue pharmacokinetics of higher generation Gd-dendrimers over 600 to 700 minutes in order to characterize the differences in the permeability of the BTB of orthotopic and ectopic RG-2 malignant gliomas and define the upper limit of pore size within the BTB of brain tumors and peripheral tumors. We determined the differences in the permeability of the BTB of an ectopic RG-2 glioma and an orthotopic RG-2 glioma within the same rat at the same time. For each animal, RG-2 glioma cells were inoculated in the right anterior brain, which was the orthotopic site, and the left temporalis muscle, which was the ectopic site. The change in blood and tumor tissue Gd concentration, a surrogate for the Gd-dendrimer concentration, was determined by calculating the molar relaxivity of the respective Gd-dendrimer generation *in vitro*, and the change in the longitudinal relaxation time before and after Gd-dendrimer bolus for each imaged volume element (voxel) *in vivo *over time.

## Methods

### PAMAM dendrimer functionalization and characterization

Bifunctional chelating agents and functionalized gadolinium-benzyl-diethyltriaminepentaacetic acid (Gd-Bz-DTPA) PAMAM dendrimers were synthesized according to procedures previously described[22]. With a molar reactant ratio of = 2:1 bifunctional chelate to dendrimer surface amine groups, isothiocyanate activated DTPA was reacted with the amine groups for 48 hours. Gadolinium was then chelated after the removal of the *t*-butyl protective groups on the DTPA. The percent by mass of Gd in each Gd-dendrimer generation was determined by elemental analysis to be: Gd-G5 (13.2%), Gd-G6 (13.0%), Gd-G7 (12.3%), and Gd-G8 (11.9%). Gd-G5 and Gd-G6 dendrimer molecular weights were determined by matrix assisted laser desorption/ionization time-of-flight (MALDI TOF) mass spectroscopy (Scripps Center for Mass Spectrometry, La Jolla, CA). Gd percent by mass of the Gd-dendrimer, in its solid form, was determined with the inductively coupled plasma-atomic emission spectroscopy (ICP-AES) method (Desert Analytics, Tucson, AZ). Gd-dendrimer infusions were normalized to 100 mM with respect to Gd.

### *In vitro *scanning transmission electron microscopy

For *in vitro *transmission electron microscopy (TEM) experiments, a 5 μL droplet of phosphate-buffer saline solution containing a sample of either Gd-G5, Gd-G6, Gd-G7 or Gd-G8 dendrimers was adsorbed onto a 3 nm-thick carbon support film covering lacey carbon electron microscopy grids. After adsorption for 2 minutes, the grids were blotted with filter paper to remove excess solution, washed 5 times with 5 μL aliquots of deionized water, and left to dry in air. Annular dark-field (ADF) scanning transmission electron microscopy (STEM) images of the Gd-dendrimers were recorded using a Tecnai TF30 electron microscope (FEI, Hillsboro, OR, USA) equipped with a Schottky field-emission gun and an in-column ADF detector (Fischione, Export, PA, USA). Molecular weight measurements of Gd-G7 and Gd-G8 dendrimers were performed with a combination STEM and energy-filtered TEM (EFTEM) imaging approach[24,25].

### *In vitro *magnetic resonance imaging for calculations of Gd-dendrimer molar relaxivity

From each of the Gd-dendrimer stock solutions to be used for *in vivo *imaging, 20 μL of Gd-dendrimer was withdrawn and diluted in 200 μL microfuge tubes containing PBS. The final concentrations of each Gd-dendrimer generation were 0.00 mM, 0.25 mM, 0.50 mM, 0.75 mM and 1.00 mM concentrations with respect to Gd. As an external control, Magnevist (Bayer, Toronto, Canada), a form of Gd-DTPA, was also diluted in 200 μL microfuge tubes containing PBS at the above concentrations. The microfuge tubes were secured in level and upright positions within a plastic container filled with deionized ultra pure water. The container was placed in a 7 cm small animal solenoid radiofrequency coil (Philips Research Laboratories, Hamburg, Germany), which was then centered within a 3.0 tesla MRI scanner (Philips Intera; Philips Medical Systems, Andover, MA). Gd signal intensity measurements were made using a series of *T*_1 _weighted spin echo sequences with identical *T*_E _(echo time, 10 ms) but different *T*_R _(repetition times; 100 ms, 300 ms, 600 ms, and 1200 ms). Using the measured Gd signal intensities and known *T*_R _and *T*_E _values, the equilibrium magnetization (M_0_) and the longitudinal relaxivity (1/*T*_1_) values were determined by non-linear regression (Eq. 1)[26].

(1)

The Gd-dendrimer molar relaxivities (*r*_1_) was calculated by linear regression (Eq. 2)[26].

(2)

The *in vitro *and *in vivo *Gd-dendrimer molar relaxivities were assumed to be equivalent for the purposes of this work[27].

### Orthotopic and ectopic RG-2 glioma induction and animal preparation for imaging

All animal experiments were approved by the National Institutes of Health Clinical Center Animal Care and Use Committee. Cryofrozen pathogen-free RG-2 glioma cells were obtained from the American Type Culture Collection (Rockville, MD) and cultured in sterile DME supplemented with 10% FBS and 2% penicillin-streptomycin in an incubator set at 37°C and 5% CO_2_. The anesthesia route for all animal experiments was isoflurane by inhalation with nose cone, 5% for induction and 1 to 2% for maintenance. On experimental day 0, the head of anesthetized adult male Fischer344 rats (F344) weighing 190 to 200 grams (Harlan Laboratories, Indianapolis, IN) was secured in a stereotactic frame with ear bars (David Kopf Instruments, Tujunga, CA). The right brain caudate nucleus (orthotopic RG-2 glioma)[28] and left temporalis muscle (ectopic RG-2 glioma) locations were stereotactically inoculated with 10^5 ^RG-2 glioma cells in 5 μL of sterile PBS. In each location, the cells were injected over 8 minutes, using a 10 μL Hamilton syringe with a blunt tip 32-gauge needle for the brain inoculate and a sharp tip 26-gauage needle for the temporalis muscle inoculate. On experimental days 11 to 12, brain imaging of re-anesthetized rats was performed following placement of polyethylene femoral venous cannula (PE-50; Becton-Dickinson, Franklin Lakes, NJ) for contrast agent infusion. Gd-dendrimers were infused at dose of 0.09 mmol Gd/kg.

### *In vitro *magnetic resonance imaging of RG-2 gliomas

For imaging, the animal was positioned supine, with face, head, and neck snugly inserted into a nose cone within the 7 cm small animal solenoid radiofrequency coil, which was then centered within the 3.0 tesla MRI scanner. Coronal, sagittal, and axial localizer scans were used in order to identify the coronal plane most perpendicular to the rat brain dorsum. After orienting the rat brain in the image volume, a fast spin echo *T*_2 _weighted anatomical scan was performed. Image acquisition parameters for the *T*_2 _scan were: *T*_R _of 6000 ms, *T*_E _of 70 ms, image matrix of 256 by 256, and slice thickness of 1 mm. In order to quantify contrast agent concentration during post imaging processing, two separate three-dimensional fast field echo *T*_1 _weighted scans were performed, one at a 3° low flip angle (low FA) of and the other at a 12° high flip angle (high FA). Image acquisition parameters for both scans were: *T*_R _of 8.1 ms, *T*_E _of 2.3 ms, image matrix of 256 by 256, and slice thickness of 1 mm. The low FA scan was performed over 1.67 min, without any Gd-dendrimer on board. For the high FA scans, which were the dynamic scans, the entire brain volume was acquired once every 20 seconds.

At the beginning of the first high FA scan, three to five pre-contrast brain volumes were acquired to guarantee the integrity of the *T*_1 _map without contrast agent (*T*_10_). Following acquisition of the pre-contrast brain volumes, a 0.09 mmol/kg dose of the respective Gd-dendrimer generation was infused. The Gd-dendrimer was infused as a slow bolus, over 1 minute, so that the blood pharmacokinetics of the respective Gd-dendrimer generation could be accurately measured during the early time points. The initial series of high FA dynamic scans were acquired for 15 minutes and subsequent high FA dynamic scans were acquired over 2 minutes at various time points. For each of the imaging sessions to acquire the Gd signal intensity data for measurement of the change in blood and tumor tissue Gd concentration over 600 to 700 minutes, the rat brains of 2 to 3 rats were imaged as frequently as possible one after the other, once every 30 to 90 minutes. For each of subsequent high FA dynamic scan, the animal was re-anesthetized and re-imaged. For each of the Gd-dendrimer generations, one additional rat head was imaged every 10 min following the initial 15 minute dynamic scan, for a total of 175 minutes, while the animal was maintained under anesthesia for the duration of the scanning session. This was to image more frequently the change in Gd signal intensity and produce voxel-by-voxel Gd concentration maps.

### Dynamic contrast-enhanced MRI data processing and analysis

Imaging data was analyzed using the Analysis of Functional NeuroImaging (AFNI; ) software suite[29]. Motion correction was performed by registering each volume of the high FA dynamic scans to the low FA scan. After volume registration, a *T*_1 _without contrast (*T*_10_) map was generated for each voxel by using the low FA signal data and the mean of the high FA dynamic scan signal data before contrast enhancement from the Gd-dendrimer bolus was visualized on the high FA dynamic scan (Eq. 3)[26].

(3)

After generating the *T*_10 _map, a *T*_1 _map was generated for each voxel of each dynamic image of each high FA dynamic scan data set after the contrast enhancement. For the high FA scan data of the 2 minute scan sessions, the average Gd signal intensity data from the 6 dynamic scans was used for the *T*_1 _map calculation. Using the *T*_10 _and *T*_1 _signal intensity map values, in addition to the Gd-dendrimer molar relaxivity value, each Gd signal data set was converted to a Gd concentration space data set (Eq. 2).

To determine the Gd concentration in the blood and RG-2 gliomas, blood and tumor voxels, respectively, were selected on coronal images of the high FA dynamic scan data sets. The Gd concentration in blood was determined in the common carotid arteries, since these were the largest caliber brain vessels in the imaging field-of-view. From within the common carotid arteries, 5 to 10 voxels that had physiologically reasonable blood *T*_10 _values of approximately 1100 ms were selected. To determine the change in blood Gd concentration over time the selected blood voxels were identified on the co-registered high FA dynamic scan data sets of the subsequent time points. The average blood Gd concentration values were then calculated for each time point.

To determine the Gd concentration in orthotopic and ectopic RG-2 gliomas, tumor tissue voxels were selected by identifying the respective tumors on the *T*_2 _weighted anatomical scans in addition to the pattern of positive contrast enhancement within the tumor tissue extravascular space on one of the 2 minute high FA dynamic scan data sets acquired between 175 and 225 minutes, since this was the time frame of maximal contrast enhancement within the tumor tissue extravascular space for Gd-G5, Gd-G6, and Gd-G7 dendrimer animal groups. For the Gd-G8 animal group, although there was no significant positive contrast enhancement within the tumor tissue extravascular space on the dynamic scan data sets, the outline of the positive contrast enhancement within the tumor microvasculature on one of the dynamic scan data sets acquired between 175 and 225 minutes was sufficient to identify tumor tissue. The selected orthotopic and ectopic RG-2 glioma tumor tissue voxels represented the respective whole tumor volumes. To determine the change in Gd concentration over time, the whole tumor volumes were then identified on the co-registered high FA dynamic scan data sets of the other time points. The average whole tumor Gd concentration values were then calculated for each time point.

For each Gd-dendrimer generation, the average Gd concentrations obtained from the common carotid arteries, the orthotopic RG-2 glioma, and the ectopic RG-2 glioma were plotted over time using Matlab (Version 7.1; The MathWorks Inc, Natick, MA). The pharmacokinetics of Gd-dendrimers in blood were qualitatively assessed due to limited number of voxels available from the common carotid artery for analysis in the context of the known limitations of dynamic contrast-enhanced MRI-based acquisition of arterial input functions.

It was possible to quantify the pharmacokinetics of Gd-dendrimer generations in tumor tissues over 600 to 700 minutes. Best fit curves were calculated using the Matlab Curve Fitting Toolbox (Version 1.1.4; The MathWorks Inc) using a bi-exponential function (Eq. 4).

(4)

where

[*Gd*]_*t *_= predictive Gd concentration at time *t *min (mM)

*a *(mM), *b *(min^-1^), *c *(mM), *d *(min^-1^) = parameters to be determined for best fit

The first term, *ae*^*bt*^, represents the fast initial exponential rise in Gd concentration and the second term, *ce*^*dt*^, represents the slow subsequent exponential decay in Gd concentration over time. The 95% confidence intervals (CI) and the root mean squared errors (RMSE) for the orthotopic and ectopic RG-2 glioma Gd concentration curve profiles were calculated.

## Results

### Physical properties of naked PAMAM and Gd-PAMAM dendrimer generations

The physical properties of naked PAMAM dendrimers (Starburst G5-G8, ethylenediamine core; Sigma-Aldrich, St. Louis, MO) and Gd-DTPA functionalized PAMAM dendrimers were characterized. Within each dendrimer generation, the amount of increase in the molecular weight between the naked dendrimer and the functionalized dendrimer is proportional to the percent conjugation of Gd-DTPA (Table 1). For each successively higher dendrimer generation, the percent conjugation of Gd-DTPA is lower due to greater steric hindrance encountered in the chelation reaction process (Table 1). The Gd-dendrimer molar relaxivities, which are the constants of proportionality required for calculation of Gd concentration from Gd signal intensity, ranged between 9.81 and 10.05 1/mM*s (Table 1).

**Table 1:** Physical properties of PAMAM and Gd-PAMAM dendrimers

Dendrimer generation (G)	Terminal amines (#)	Naked PAMAM molecular weight # (kDa)	Gd-PAMAM dendrimer molecular weight (kDa)	Gd-DTPA conjugation (%)	Molar relaxivity&(1/mM*s)
G5	128	29	79†	52	9.81
G6	256	58	138†	45	10.04
G7	512	116	283‡	43	9.82
G8	1024	233	490‡	36	10.05

#molecular weight obtained from Dendritech, Inc.

†molecular weight measured by MALDI TOF MS

‡mean molecular weight measured by ADF STEM and EFTEM

&molar relaxivity of Gd-DTPA measured to be 4.13 1/mM*s

**Figure 1:**
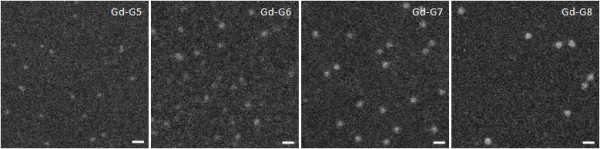
**Transmission electron microscopy of higher generation Gd-dendrimers**. Annular dark-field scanning transmission electron microscopy (ADF STEM) images of unstained Gd-G5, Gd-G6, Gd-G7, and Gd-G8 dendrimers adsorbed onto an ultrathin carbon support film. The diameters of one hundred Gd-G7 and Gd-G8 dendrimers were measured. Scale bar = 20 nm.

ADF STEM of Gd-G5 through Gd-G8 dendrimers demonstrated uniformity in particle shape and size within any particular Gd-dendrimer generation (Figure 1). ADF STEM confirmed a small increase of approximately 2 nm in particle diameter between successive generations (Figure 1). The masses of Gd-G7 and Gd-G8 dendrimers were sufficient that the sizes and molecular weights of these Gd-dendrimer generations could be measured by ADF STEM and STEM-EFTEM, respectively. The molecular weights and diameters of one hundred Gd-G7 and Gd-G8 dendrimers were measured. The average molecular weight of Gd-G7 was 283 ± 5 kDa and that of Gd-G8 dendrimers was 490 ± 5 kDa (mean ± standard error of the mean) (Table 1). The average diameter of Gd-G7 dendrimers was 10.9 ± 0.7 nm and that of Gd-G8 dendrimers was 12.7 ± 0.7 nm (mean ± standard deviation).

### Permeability of the BTB of orthotopic and ectopic RG-2 gliomas to Gd-PAMAM dendrimer generations

**Figure 2:**
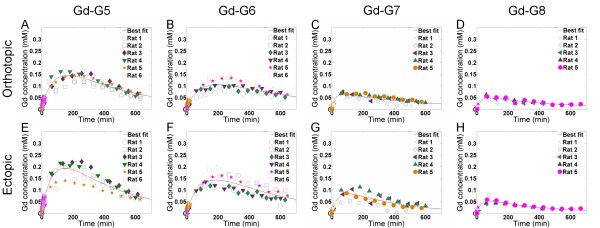
**Pharmacokinetics of Gd-dendrimer generations in orthotopic RG-2 gliomas and ectopic RG-2 gliomas over 600 to 700 minutes**. Respective Gd-dendrimer generation was intravenously infused over 1 minute (0.09 mmol Gd/kg) during the initial 15 minute dynamic contrast-enhanced MRI scan session. Subsequent dynamic scan sessions of re-anesthetized animals were conducted at 30 to 90 minute time intervals. Whole tumor tissue Gd concentrations for the orthotopic and ectopic RG-2 gliomas were calculated for each of the dynamic scan session time points. Shown is the change in the Gd concentration of respective Gd-dendrimer generations in orthotopic RG-2 gliomas and ectopic RG-2 gliomas over 600 to 700 minutes. Superimposed is the best fit curve Gd concentration curve for the respective Gd-dendrimer generations. Panels A through D are orthotopic glioma Gd concentrations over time. Panels E through H are ectopic glioma Gd concentrations over time A. Gd-G5 (Orthotopic, N = 6), B. Gd-G6 (Orthotopic, N = 6), C. Gd-G7 (Orthotopic, N = 5), D. Gd-G8 (Orthotopic, N = 5), E. Gd-G5 (Ectopic, N = 6), F. Gd-G6 (Ectopic, N = 6), G. Gd-G7 (Ectopic, N = 5), H. Gd-G8 (Ectopic, N = 5).

Gd-G5 dendrimers extravasated across the BTB of both orthotopic and ectopic RG-2 gliomas and accumulated within the respective tumor tissue extravascular spaces (Figure 2, panels A and E). However, the Gd-G5 dendrimers extravasated to a lesser extent across the BTB of orthotopic RG-2 gliomas than the BTB of ectopic RG-2 gliomas indicating the BTB of orthotopic RG-2 gliomas was less permeable than the BTB of ectopic RG-2 gliomas. Thus, the peak Gd concentration of Gd-G5 dendrimers in orthotopic tumors was 0.147 mM, whereas the peak Gd concentration of Gd-G5 dendrimers in ectopic tumors was 0.195 mM (Table 2, Additional file 1).

**Table 2:** Gd-PAMAM dendrimer peak concentrations in orthotopic RG-2 gliomas versus ectopic RG-2 gliomas*

Gd-dendrimer generation (G)	Peak concentration in orthotopic RG-2 gliomas (mM)	Peak concentration time point (min)	Peak concentration in ectopic RG-2 gliomas (mM)	Peak concentration time point (min)
Gd-G5	0.147	167	0.195	149
Gd-G6	0.106	200	0.144	189
Gd-G7	0.064	75	0.084	107
Gd-G8	0.049	77	0.052	81

*95% confidence intervals (CI) and root mean squared errors (RMSE) for best fit curve concentrations from the bi-exponential function [*Gd*]_*t *_= *ae*^*bt*^*+ ce*^*dt *^are reported in Additional file 1

Gd-G6 dendrimers also extravasated across the BTB of both orthotopic and ectopic RG-2 gliomas and accumulated within the respective tumor tissue extravascular spaces (Figure 2, panels B and F). Gd-G6 dendrimers accumulated to lesser extent than Gd-G5 dendrimers in both orthotopic and ectopic tumor tissue extravascular spaces. As was the case for Gd-G5 dendrimers, the Gd-G6 dendrimers extravasated to a lesser extent across the BTB of orthotopic RG-2 gliomas than the BTB of ectopic RG-2 gliomas, once again indicating the BTB of orthotopic RG-2 gliomas was less permeable than the BTB of ectopic RG-2 gliomas. Thus, the peak Gd concentration of Gd-G6 dendrimers in orthotopic tumors was 0.106 mM, whereas the peak Gd concentration of Gd-G6 dendrimers in ectopic tumors was 0.144 mM.

Gd-G7 dendrimers minimally extravasated across the BTB of both orthotopic and ectopic RG-2 gliomas and so minimally accumulated within the respective tumor tissue extravascular spaces (Figure 2, panels C and G). Gd-G7 dendrimers accumulated to an even lesser extent than Gd-G6 dendrimers in both orthotopic and ectopic tumor tissue extravascular spaces. As was the case for Gd-G6 dendrimers, the Gd-G7 dendrimers extravasated to a lesser extent across the BTB of orthotopic RG-2 gliomas than the BTB of ectopic RG-2 gliomas, once again indicating the BTB of orthotopic RG-2 gliomas was less permeable than the BTB of ectopic RG-2 gliomas. Thus, the peak Gd concentration of Gd-G7 dendrimers in orthotopic tumors was 0.064 mM, whereas the peak Gd concentration of Gd-G7 dendrimers in ectopic tumors was 0.084 mM (Table 2, Additional file 1).

Gd-G8 dendrimers did not extravasate across the BTB of orthotopic and ectopic RG-2 gliomas. The change in Gd concentration over time for both orthotopic and ectopic RG-2 gliomas was similar (Figure 2, panels D and H). The peak Gd concentrations of Gd-G8 dendrimers in both orthotopic and ectopic tumors were similar: the peak Gd concentration of Gd-G8 dendrimers in orthotopic tumors was 0.049 mM and that in ectopic tumors was 0.052 mM (Table 2, Additional file 1). The peak Gd concentrations in orthotopic and ectopic tumors reflect the peak Gd-G8 dendrimer concentrations within the microvasculature of the respective tumors and not the extravascular tumor tissue space.

### Physiologic upper limit of pore size within the BTB of orthotopic and ectopic RG-2 gliomas as visualized on Gd concentration maps

**Figure 3:**
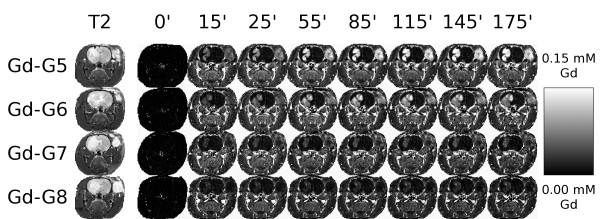
**Gd concentration maps of Gd-dendrimer contrast enhancement over 175 minutes**. For one additional animal in each Gd-dendrimer generation group the respective Gd-dendrimer generation was intravenously infused over 1 minute (0.09 mmol Gd/kg) while the animal was maintained under anesthesia for the duration of the 175 minute dynamic contrast-enhanced MRI session. Voxel-by-voxel Gd concentration maps were generated. Shown are the voxel-by-voxel Gd concentration maps for the respective Gd-dendrimer generations at the 15 minute time point and then at 30 minute time intervals thereafter. First row, Gd-G5 dendrimer (Orthotopic RG-2 glioma tumor volume, 45 mm^3^; ectopic RG-2 glioma tumor volume, 113 mm^3^). Second row, Gd-G6 dendrimer (Orthotopic RG-2 glioma tumor volume, 97 mm^3^; ectopic RG-2 glioma tumor volume, 184 mm^3^). Third row, Gd-G7 dendrimer (Orthotopic RG-2 glioma tumor volume, 53 mm^3^; ectopic RG-2 glioma tumor volume, 135 mm^3^). Fourth row, Gd-G8 dendrimer (Orthotopic RG-2 glioma tumor volume, 50 mm^3^; ectopic RG-2 glioma tumor volume, 163 mm^3^).

For each of the Gd-dendrimer generations, after the initial 15 minute dynamic scan, the orthotopic and ectopic RG-2 gliomas of one additional animal were imaged every 10 minutes for a total of 175 minutes, while the animal was under continuous anesthesia. The Gd concentration maps from selected dynamic scans of these imaging sessions are shown in Figure 3. The hemodynamic depression associated with the continuous anesthesia is reflected in the lower peak contrast enhancement observed.

Gd-G5 dendrimers readily extravasated across the BTB of both orthotopic and ectopic RG-2 gliomas and accumulated over time within the respective tumor tissue extravascular spaces, as evidenced by the significant positive contrast enhancement over time in the respective tumor tissues (Figure 3, first row). Gd-G6 dendrimers also extravasated across the BTB of both orthotopic and ectopic RG-2 gliomas and accumulated over time within the respective tumor tissue extravascular spaces (Figure 3, second row), although to a lesser extent than Gd-G5 dendrimers (Figure 3, first row).

Gd-G7 dendrimers minimally extravasated across the BTB of both orthotopic and ectopic RG-2 gliomas and so minimally accumulated over time within the respective tumor tissue extravascular spaces (Figure 3, third row). Gd-G8 dendrimers did not extravasate over time across the BTB of both orthotopic and ectopic RG-2 gliomas, but instead remained within the tumor microvasculature, as evidenced by the lack of contrast enhancement over time within the respective tumor tissue extravascular spaces (Figure 3, fourth row). Therefore, the physiologic upper limit of pore size within the BTB of both malignant brain tumors and peripheral solid tumors is equivalent. Since the diameter of our Gd-G7 dendrimers and Gd-G8 dendrimers was 10.9 ± 0.7 nm and 12.7 ± 0.7 nm (mean ± standard deviation), the upper limit of pore size within the BTB of both orthotopic RG-2 gliomas and ectopic RG-2 gliomas is approximately 12 nm.

## Discussion

In the BTB of malignant solid tumor microvasculature, the anatomic pore sizes of trans-endothelial cell fenestrations, caveolae and VVOs range between 40 nm to 200 nm[10,13,14], and the sizes of inter-endothelial cell gaps range between 100 nm and 4700 nm[10,12,13]. Irrespective of tumor host site, trans-endothelial cell fenestrations, caveolae, and VVOs are present more often than the inter-endothelial cell gaps in the BTB of malignant solid tumors[4,9,10]. Due to host site influence the BTB of peripheral tumors has more frequent trans-endothelial cell fenestrations, caveolae and VVOs, and larger inter-endothelial cell gaps than the BTB of malignant brain tumor microvasculature[6,10]. The higher permeability of the BTB of peripheral tumors than that of brain tumors has been attributed to the larger anatomic pore sizes of the inter-endothelial cell gaps[12,15]. We reasoned that in the physiologic state *in vivo *the intact luminal glycocalyx layer would be the primary impediment to the transvascular passage of even small nanoparticles across the BTB of malignant solid tumors independent of tumor host site.

In this study, with dynamic contrast-enhanced MRI we imaged the blood and tumor tissue pharmacokinetics of intravenously infused Gd-PAMAM dendrimer nanoparticles G5 through G8 over 600 to 700 minutes. We compared the permeability of the BTB of RG-2 gliomas grown within the brain, the orthotopic site, to that of the BTB of RG-2 gliomas grown outside the brain in the temporalis skeletal muscle, the ectopic site. We used this animal model to characterize the differences in the permeability of the BTB of a malignant brain tumor to that of the BTB of a peripheral solid tumor, and to define the upper limit of pore size within the BTB of the respective solid tumors. Using this approach, we found that the physiologic upper limit of pore size in the BTB of brain RG-2 gliomas and peripheral RG-2 gliomas is approximately 12 nm.

In the case of brain RG-2 gliomas, we report here that the physiologic upper limit of pore size in the BTB of orthotopic RG-2 gliomas growing in brain tissue is approximately 12 nm. Our present finding is in agreement with our previously reported finding that the upper limit of pore size in the BTB of orthotopic RG-2 gliomas is approximately 12 nm[22]. Both in our prior and present work, we probed the upper limit of the pore size within the BTB with dynamic contrast-enhanced MRI using successively higher generation Gd-DTPA labeled PAMAM dendrimer nanoparticles with a neutralized particle exterior. The positive charge on exterior of the naked PAMAM dendrimer generations was neutralized by the conjugation of Gd-DTPA (charge -2) to approximately 40% to 50% of the terminal amines on the exterior. Therefore, the Gd-DTPA labeled dendrimer generations that were used for this study would have not been toxic to the negatively charged glycocalyx overlaying the endothelial cells of the BTB.

In the case of peripheral RG-2 gliomas, we report here that the physiologic upper limit of pore size in the BTB of ectopic RG-2 gliomas growing in skeletal muscle is equivalent to the upper limit of pore size in the BTB of orthotopic RG-2 gliomas growing in brain tissue, and is also approximately 12 nm. The physiologic upper limit of pore size in the BTB of peripheral RG-2 gliomas that we report here is significantly lower than what has been previously reported[15]. In the past, the physiologic upper limit of the pore size within the BTB of orthotopic and ectopic malignant peripheral tumors has been probed by intra-vital fluorescence microscopy 24 hours after the intravenous infusion of liposomes and microspheres with a cationic exterior, and it has been reported the upper limit of the pore size within the BTB of peripheral tumors is between 200 nm and 1200 nm[15]. This higher upper limit of pore size would be most likely due to the toxicity of the cationic liposomes and microspheres to the negatively charged glycocalyx overlaying the endothelial cells of the BTB. The circulation of cationic particles for 24 hours would be sufficient time to expose the underlying smaller-sized trans-endothelial cell fenestrations and VVOs as well as the larger-sized inter-endothelial cell gaps. The transvascular extravasation of the particles across the exposed inter-endothelial cell gaps into the tumor tissue extravascular space, or alternatively, entrapment in the peri-vascular space along the basement membrane would result in the over-estimation of the actual physiologic upper limit of pore size within the BTB.

**Figure 4:**
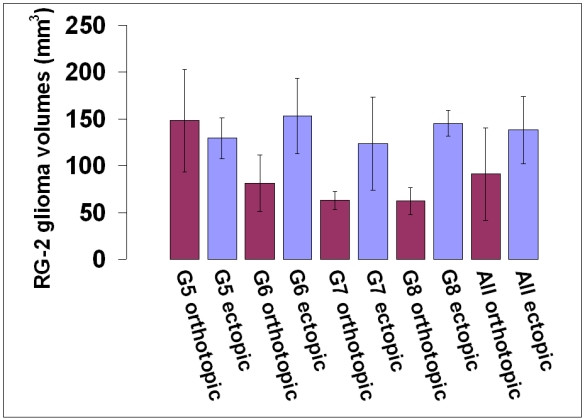
**Tumor volumes of orthotopic and ectopic RG-2 gliomas of each Gd-dendrimer generation**. Whole tumor tissue volumes, in mm^3^, were determined for the orthotopic and ectopic RG-2 gliomas of each of the Gd-dendrimer generation groups using the *T*_2 _weighted anatomical scans and dynamic contrast-enhanced MRI data sets as described in the Methods section. Shown are the average whole tumor volumes of orthotopic and ectopic RG-2 gliomas of each Gd-dendrimer generation. A. Gd-G5 (Orthotopic, N = 6; Ectopic, N = 6), B. Gd-G6 (Orthotopic, N = 6; Ectopic, N = 6), C. Gd-G7 (Orthotopic, N = 5; Ectopic, N = 5), D. Gd-G8 (Orthotopic, N = 5; Ectopic, N = 5). Error bars represent standard deviation.

We found that Gd-G5, Gd-G6, and Gd-G7 dendrimers extravasated across the BTB of ectopic RG-2 gliomas as well as that of orthotopic RG-2 gliomas. However, these Gd-dendrimer generations extravasated to a greater extent across the BTB of ectopic RG-2 gliomas than the BTB of orthotopic RG-2 gliomas, as Gd-G5, Gd-G6, and Gd-G7 dendrimers achieved higher peak concentrations in the tumor tissue extravascular space of ectopic RG-2 malignant gliomas than in the tumor tissue extravascular space of orthotopic RG-2 malignant gliomas. Based on these findings, the BTB of the ectopic RG-2 malignant gliomas is more permeable than the BTB of orthotopic RG-2 malignant gliomas. The observed higher permeability of the BTB of ectopic RG-2 gliomas in this animal model may be in part due to host site dependent differences in tumor volume, since the tumor volumes of the ectopic RG-2 gliomas where generally larger than those of the orthotopic RG-2 gliomas (Figure 4). Although this may be the case, the higher permeability of BTB of ectopic RG-2 gliomas compared to that of the BTB of orthotopic RG-2 gliomas is consistent with the reported higher permeability of the BTB of malignant peripheral tumors compared to that of the BTB of malignant brain tumors[5,7].

With each successively higher Gd-dendrimer generation there was an approximately 2 nm increase in Gd-dendrimer diameter. Although there were relatively small increases in Gd-dendrimer particle sizes, there were significant decreases in particle extravasation across the BTB with increasing Gd-dendrimer generation, irrespective of RG-2 glioma host site. Gd-G7 dendrimers extravasated only minimally across the BTB, and the Gd-G8 dendrimers were large enough that these particles did not extravasate across either the BTB of ectopic RG-2 gliomas or that of orthotopic RG-2 gliomas. As a result, Gd-G8 dendrimers did not accumulate over time in the respective tumor tissue extravascular spaces, and instead remained in the tumor microvasculature. The peak Gd concentrations of Gd-G8 dendrimers in ectopic RG-2 gliomas and orthotopic RG-2 gliomas were similar and reflect the peak Gd-G8 dendrimer concentrations within the microvasculature of the respective tumors.

**Figure 5:**
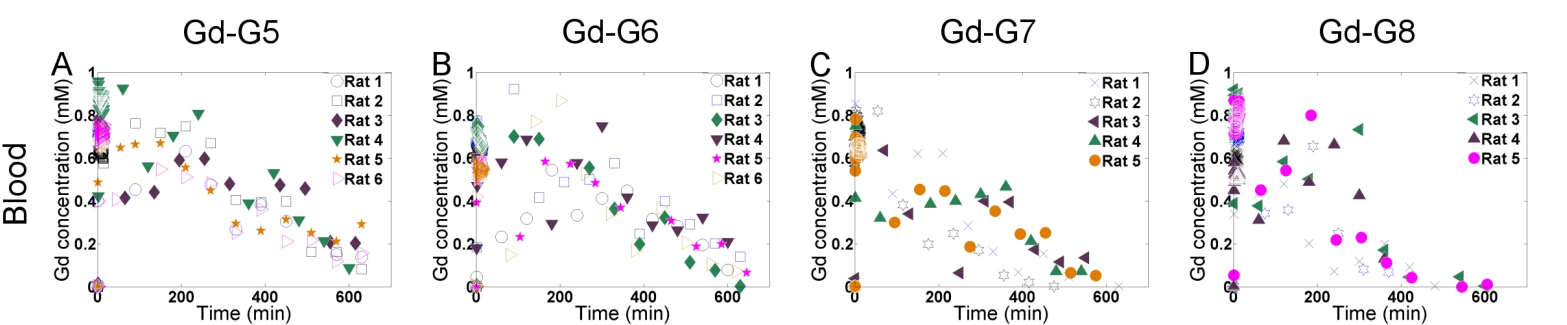
**Blood pharmacokinetics of Gd-dendrimer generations over 600 to 700 minutes**. Five to ten voxels were selected from within the common carotid arteries. For the selected voxels, the average blood Gd concentrations were determined for each of the dynamic scan session time points. Shown is the change in average blood Gd concentration of the respective Gd-dendrimer generations over 600 to 700 minutes. A. Gd-G5 (N = 6), B. Gd-G6 (N = 6), C. Gd-G7 (N = 5), D. Gd-G8 (N = 5).

We found that the blood half-lives of Gd-G5 and Gd-G6 dendrimers to be longer than those of Gd-G7 and Gd-G8 dendrimers (Figure 5). In case of Gd-G5 and Gd-G6 dendrimers, the relatively longer blood half-lives are due to the sizes of these Gd-dendrimer generations being large enough to evade kidney filtration following transvascular extravasation across the discontinuous microvasculature of the glomeruli of the kidneys[30], yet small enough to evade liver and spleen reticuloendothelial system opsonization following transvascular extravasation across the discontinuous microvasculature of the liver and spleen[31]. Therefore, Gd-G5 and Gd-G6 dendrimers were not effectively cleared from blood circulation and had longer blood half-lives than Gd-G7 and Gd-G8 dendrimers. In the case of Gd-G7 and Gd-G8 dendrimers, due to the relatively few number of voxels available for analysis and the finite sensitivity of dynamic contrast-enhanced MRI-based analysis, it was not possible to accurately detect the relatively small changes in blood Gd concentration at the latter imaging time points when the Gd-G7 and Gd-G8 dendrimer generations had been cleared from the blood circulation (Figure 5, panels C and D). However, it was possible to qualitatively assess the differences in the blood half-lives of Gd-G7 and Gd-G8 dendrimers compared to those of the Gd-G5 and Gd-G6 dendrimers. The blood half-lives of Gd-G7 and Gd-G8 dendrimers were shorter than those of the Gd-G5 and Gd-G6 dendrimers likely due to the sizes of these Gd-dendrimers being too large to evade opsonization by reticuloendothelial system of the liver and spleen[31]. Even though Gd-G7 dendrimers were small enough to extravasate across the BTB and Gd-G8 dendrimers were too large to extravasate across the BTB, both Gd-G7 and Gd-G8 dendrimers were effectively cleared from blood circulation and had shorter blood half-lives than Gd-G5 and Gd-G6 dendrimers. These findings suggest that nanoparticles within the size range of Gd-G5 and Gd-G6 dendrimers would be both permeable to the BTB of malignant solid tumor microvasculature and also possess blood half-lives sufficiently long to allow for particles to effectively accumulate over time within the tumor tissue extravascular space by the enhanced permeation and retention (EPR) effect[32].

Since the sizes of hydrated dendrimer generations, measured by small-angle X-ray scattering (SAXS)[33] and small-angle neutron scattering (SANS)[34], are similar to the sizes of respective dehydrated and stained dendrimer generations measured by TEM[35], here we used ADF STEM to the measure the sizes of the Gd-G7 dendrimers and Gd-G8 dendrimers dried on ultrathin carbon support film[24,25]. We found the diameters of the Gd-G7 dendrimers to be 10.9 ± 0.7 nm and those of the Gd-G8 dendrimers to be 12.7 ± 0.7 nm (mean ± standard deviation). Since Gd-G7 dendrimers were permeable to both the BTB of ectopic RG-2 gliomas and orthotopic RG-2 gliomas, but the Gd-G8 dendrimers were not, this establishes the effective physiologic upper limit of pore size in both the BTB of ectopic RG-2 gliomas and orthotopic RG-2 gliomas as being approximately 12 nm.

The previously reported higher physiologic upper limit of pore size in the BTB of malignant solid tumors, based on intra-vital fluorescence microscopy of tumor tissue 24 hours following the intravenous infusion of cationic nanoparticles, appears to have been a gross over-estimation of the actual physiologic upper limit of pore size. The most plausible explanation for this is that the positively charged exterior of the cationic nanoparticles was toxic to the negatively charged glycocalyx surface coat of the BTB. We report here, based on dynamic contrast-enhanced MRI of tumor tissue following the intravenous infusion of neutralized nanoparticles, that the physiologic upper limit of pore size is much lower, being approximately 12 nm, when the luminal fibrous glycocalyx of the BTB is maintained intact.

The ultrastructure of the glycocalyx has been previously investigated in frog mesentery capillaries since the morphology of this type of microvasculature is similar to that of mammalian microvasculature of the continuous type, for example that of skeletal muscle[36,37]. In such continuous microvasculature, there are small pores in the endothelial barrier underlying the glycocalyx that allow for the minimal transvascular extravasation of macromolecules smaller than 4 to 5 nm in diameter across the barrier[38,39]. It has been reported that when the fibrous meshwork of the glycocalyx layer overlaying these small pores is enzymatically degraded, then there is an increase in the transvascular extravasation of macromolecules across the endothelial barrier[40,41] even though there are no accompanying anatomic changes in the underlying pores[41]. Based on such work, it would be reasonable to speculate that the observed increase in transvascular extravasation of macromolecules across the endothelial barrier of continuous microvasculature is a result of an increase in the physiologic upper limit of pore size in the barrier due to the disruption of the glycocalyx layer. The damage that occurs to the glycocalyx of the endothelial barrier of continuous microvasculature following enzymatic degradation would be analogous to that which occurs to the glycocalyx of the BTB of malignant tumor microvasculature following prolonged exposure to the positive exterior of cationic particles.

In the case of the BTB of malignant solid tumor microvasculature, we report here that in the physiologic state *in vivo *that only particles smaller than approximately 12 nm in diameter can effectively extravasate across the BTB independent of tumor location. Although we found that the physiologic upper limit of pore size in the BTB of brain tumors (orthotopic RG-2 gliomas) as well as peripheral tumors (ectopic RG-2 gliomas) was equivalent, the transvascular extravasation of the permeable particles (i.e. Gd-G5, Gd-G6, and Gd-G7 dendrimers) was greater across the BTB of the peripheral tumors. Even though in this work we did not study the ultrastructure of the glycocalyx of the BTB of brain and peripheral tumor microvasculature, we suspect that there are similarities in the arrangement and spacing of the glycocalyx fibers overlaying the pores within the BTB of brain and peripheral tumor microvasculature. This would account for the physiologic upper limit of pore size in the BTB of malignant solid tumor microvasculature being equivalent and independent of tumor location. The higher permeability of the BTB of malignant peripheral tumors to macromolecules, in this case the Gd-G5, Gd-G6 and Gd-G7 dendrimer nanoparticles, may then be explained by the presence of more pores underlying the glycocalyx, which would allow for the transvascular extravasation of greater numbers of particles smaller than approximately 12 nm in diameter.

## Conclusion

We report here that the physiologic upper limit of pore size in the BTB of malignant solid tumor microvasculature is approximately 12 nanometers. Since in the physiologic state *in vivo *the fibrous glycocalyx overlays the luminal surface of the BTB of both brain tumor and peripheral tumor microvasculature, the physiologic upper limit of pore size in the BTB of malignant solid tumor microvasculature is equivalent and independent of tumor host site. The higher permeability of malignant peripheral tumor microvasculature to macromolecules smaller than approximately 12 nm in diameter is attributable to the presence of a greater number of pores underlying the glycocalyx of the BTB of peripheral tumor microvasculature.

## Competing interests

The authors declare that they have no competing interests.

## Authors' contributions

HS conceptualized and designed overall study; performed MRI experiments, analyzed MRI data, interpreted overall study results, and wrote the manuscript. ASK assisted with MRI experiments, data analysis, and figure preparation. HW synthesized functionalized dendrimers. AAS characterized functionalized dendrimers with electron microscopy. CMW assisted with functionalized dendrimer synthesis. MAA assisted with electron microscopic dendrimer characterization. GLG supervised synthesis of the functionalized dendrimers. RDL supervised characterization of functionalized dendrimers with electron microscopy. HV assisted with MRI experiments, data analysis, and figure preparation. All authors read and proofed the final manuscript.

## Supplementary Material

Additional file 1 **95% confidence intervals (CI) and root mean squared errors (RMSE) for best fit curve concentrations from the bi-exponential function [*Gd*]_*t *_= *ae*^*bt*^+ *ce*^*dt*^**. The data in the table represent the statistical analysis for the orthotopic and ectopic RG-2 glioma Gd concentration curve profiles for the respective Gd-dendrimer generations over 600 to 700 minutes. A best fit was established for each Gd concentration curve profile as indicated by the corresponding low RMSE value. Note: 1 RMSE per profile. Click here for file
