# Supplementary material for: Physiologic upper limit of pore size in the blood-tumor barrier of malignant solid tumors
Source: J Transl Med. 2009 Jun 23;7:51. doi: 10.1186/1479-5876-7-51 (PMC2706803; doi:10.1186/1479-5876-7-51)
Supplement: Additional file 1 — 95% confidence intervals (CI) and root mean squared errors (RMSE) for best fit curve concentrations from the bi-exponential function [Gd]t = aebt+ cedt. The data in the table represent the statistical analysis for the orthotopic and ectopic RG-2 glioma Gd concentration curve profiles for the respective Gd-dendrimer generations over 600 to 700 minutes. A best fit was established for each Gd concentration curve profile as indicated by the corresponding low RMSE value. Note: 1 RMSE per profile. [file 1479-5876-7-51-S1.doc]

## Additional file 1 - 95% confidence intervals (CI) and root mean squared errors (RMSE) for best fit curve concentrations from the bi-exponential function [*Gd*]*_t_* = *ae^bt^ + ce^dt^*

| Gd-dendrimer | RG-2 glioma | *a* (mM); 95% CI | *b* (min^-1^); 95% CI | *c* (mM); 95% CI | *d* (min^-1^); 95% CI | RMSE^ |
| --- | --- | --- | --- | --- | --- | --- |
| Gd-G5 | Orthotopic | -0.246  (-0.285,-0.207) | -0.0123  (-0.0144,-0.0102) | 0.256  (0.216, 0.296) | -0.00220  (-0.00255,-0.00185) | 0.010 |
|  | Ectopic | -0.330  (-0.375,-0.286) | -0.0139  (-0.0160,-0.0119) | 0.340  (0.294, 0.385) | -0.00247  (-0.00280,-0.00215) | 0.013 |
| Gd-G6 | Orthotopic | -0.167  (-0.210,-0.124) | -0.0102  (-0.0130,-0.00749) | 0.180  (0.136,0.224) | -0.00173  (-0.00221,-0.00124) | 0.010 |
|  | Ectopic | -0.260  (-0.326,-0.194) | -0.0101  (-0.0125,-0.00779) | 0.272  (0.204, 0.339) | -0.00216  (-0.00264,-0.00167) | 0.011 |
| Gd-G7 | Orthotopic | -0.0658  (-0.0723,-0.0592) | -0.0437  (-0.0504,-0.0371) | 0.0744  (0.0678, 0.0810) | -0.00165  (-0.00194,-0.00136) | 0.006 |
|  | Ectopic | -0.118  (-0.145,-0.0909) | -0.0206  (-0.0264,-0.0149) | 0.130  (0.102, 0.157) | -0.00276  (-0.00342,-0.00210) | 0.011 |
| Gd-G8 | Orthotopic | -0.0503  (-0.0556,-0.0450) | -0.0389  (-0.0452,-0.0326) | 0.05939  (0.0540, 0.0648) | -0.001906  (-0.00222,-0.00159) | 0.005 |
|  | Ectopic | -0.0563  (-0.0659,-0.0468) | -0.0316  (-0.0392,-0.0240) | 0.0685  (0.0587, 0.0783) | -0.00244  (-0.00295,-0.00193) | 0.007 |

^Root mean squared error (mM)
